# Zika virus-specific and orthoflavivirus-cross-reactive IgGs correlate with Zika virus seroneutralization depending on prior dengue virus infection

**DOI:** 10.1371/journal.pntd.0013274

**Published:** 2025-07-09

**Authors:** Solène Marquine, Sébastien Briolant, Damien Claverie, Jessica Denis, Manon Geulen, Laurent Bosio, Bernard Tenebray, Sarah Attoumani, Annabelle Garnier, Laurence Cheutin, Gilda Grard, Bruno Coutard, Guillaume A. Durand, Cyril Badaut

**Affiliations:** 1 Unité de Virologie, Institut de Recherche Biomédicale des Armées (IRBA), Marseille, France; 2 Unité des Virus Émergents (UVE: Aix-Marseille Univ, Università di Corsica, Inserm, France; 3 Unité de Parasitologie et Entomologie, Institut de Recherche Biomédicale des Armées, Marseille, France; 4 Aix Marseille Université, SSA, AP-HM, RITMES, Marseille, France; 5 IHU Méditerranée Infection, Marseille, France; 6 Unité de Neurophysiologie du Stress, Institut de Recherche Biomédicale des Armées, 1 place du Général Valérie André, Brétigny-sur-Orge Cedex, France; 7 Unité Interaction Hôte-Pathogènes, Institut de Recherche Biomédicale des Armées, Brétigny sur Orge, France; 8 Centre National de Référence des Arbovirus, Inserm-IRBA, Marseille, France; 9 Unité de Virologie, Institut de Recherche Biomédicale des Armées, Brétigny sur Orge, France; Colorado State University, UNITED STATES OF AMERICA

## Abstract

Due to their expanding geographical distribution, many orthoflaviviruses co-circulate, increasing the likelihood of serial infections in individuals living in endemic areas. The consequences of such infection histories remain poorly understood. Using a cohort of Zika virus-infected patients, we modeled the kinetics of individual humoral responses to Zika virus (ZIKV) infection. We compared them between patients with and without prior orthoflavivirus exposure. We determined the day the humoral response was maximal and the maximum amplitude. We then determined the characteristic values of specific and cross-reactive responses for each individual, whether previously infected with dengue virus (DENV) or not. ZIKV neutralization by sera from ZIKV-infected individuals was mainly attributable to virus-specific antibodies. However, the same sera were able to neutralize all four DENV serotypes. ZIKV neutralization by sera from patients with prior DENV infection was mainly due to cross-reactive antibodies, which were also able to seroneutralize all four DENV serotypes. The de-novo antibody response induced against ZIKV was masked by the anamnestic response against DENV. Overall, our results show that the humoral response to an orthoflavivirus infection is influenced by the patient’s history of prior orthologous orthoflavivirus infections.

## Author summary

Zika and dengue viruses are closely related and often spread in the same regions of the world. Due to their similarities, the immune system produces antibodies that react to both viruses. However, other antibodies are specific and only recognize one virus. A cohort of patients who had recently contracted the Zika virus was monitored. It is noteworthy that a subset of these individuals had previously encountered infection with the dengue virus. Zika virus-infected patients demonstrated the production of both Zika virus-specific and orthoflaviviruses-specific antibodies. Patients with previous dengue virus infections primarily used immune “memory” from past exposure, leading to greater production of antibodies that react to related orthoflaviviruses. Their antibodies neutralized not only Zika virus but also dengue virus. This suggests that the immune response to Zika virus can be shaped, and sometimes masked, by previous infection with similar viruses such as dengue virus. These results highlight how pre-existing immunity can strongly shape the body’s response to new infections. This has important consequences for improving vaccines and treatments, and for developing more accurate diagnostic tools.

## Introduction

Zika virus (ZIKV), discovered in 1947 in Uganda [1], has caused epidemics in French Polynesia and the Americas in recent decades [2,3]. ZIKV is co-endemic with dengue virus (DENV) in tropical and subtropical regions worldwide. Most infections caused by these orthoflaviviruses are undetectable, with 80% of infected patients remaining asymptomatic. However, in some cases, following ZIKV infection, symptoms can be severe and include brain damage, leading to neurological complications, such as microcephaly in fetuses [4] or Guillain-Barré syndrome in adults [5].

DENV and ZIKV proteins share sequence homology that results in common epitopes, including those on the surface of the envelope [6]. Thus, a large fraction of the antibodies (Abs) directed against these orthoflaviviruses are cross-reactive. However, certain Abs recognize specific epitopes mainly exposed on the domain II fusion loop or domain III of the envelope protein [7] that contribute to the neutralization of the infecting virus [8]. The relationship between these specific IgGs and their neutralizing function has been extensively studied, in particular, in co-endemic areas where several orthoflaviviruses can repeatedly infect the population. The immune response to a prior DENV infection can either exacerbate or prevent symptoms that may be observed after subsequent heterologous DENV or ZIKV infections. Severe forms of DENV infection observed during secondary heterologous DENV or ZIKV infections have been shown to be associated with antibody-dependent enhancement and cytokine storms [9,10]. Conversely, it is known that a second DENV infection offers temporary protection against all DENV serotypes due to high concentrations of protective cross-reactive anti-DENV Abs. ZIKV cross-reactive neutralizing antibodies are non-detectable six months after DENV infection [11]. However, exposure to DENV can trigger an anamnestic response, accelerating antibody production during ZIKV infection by activating B cells initially selected during the primary DENV infection [12]. The impact of a prior DENV infection on subsequent non-DENV orthoflavivirus infections has been epidemiologically studied [13,14]. However, the impact is still unclear concerning a second orthoflavivirus infection with ZIKV, with contradictory findings reported [15,16].

DENV infection followed by ZIKV infection leads to cross-reactive protection and increases the production of long-persisting cross-reactive Abs [17]. The presence of such Abs correlates with neutralizing antibody titers [13,18].The IgG cross-reactivity observed after more than one DENV infection due to pre-existing immunity to DENV has been characterized by studies of the memory B-cell response [16,19]. Specific or cross-reactive antibody responses to a given DENV serotype, as well as their relative contribution to neutralization during the early convalescence phase after ZIKV infection, have never been directly described. DENV infection induces a cross-reactive memory B-cell response that recognizes ZIKV, in particular, after second DENV infection [19]. The avidity of Abs produced after a second infection is higher for heterologous DENV than that produced after an initial DENV infection [20]. Certain serotype-targeting neutralizing Abs (nAbs) can neutralize one or more DENV serotypes. Consequently, nAbs protect against symptomatic DENV infection depending on the infection history and infecting serotype [21]. Cross-neutralization can occur when a high concentration of cross-reacting Abs allows heterologous opsonization of DENV or when specific Abs cross-react with a neutralizing epitope [22]. Overall, patients with prior DENV infection show better immune responses to ZIKV. Epidemiological studies [23] have shown that pre-existing anti-orthoflavivirus immunity does not enhance ZIKV-related pathology, whereas animal experiments have shown the contrary [24–26].

We conducted a one-year-long longitudinal study on ZIKV-infected patients with or without a previous orthoflavivirus infection to better understand the immune response to ZIKV infection and the consequences of a former orthoflavivirus infection on this response. We thus followed the specific antibody response of Zika virus envelope protein domain III (ZEDIII)-specific antibodies. This antigen is considered to be virus-specific [22,27], carrying 90% specific epitopes [28]. Non-ZIKV-specific epitopes are detected during a second orthoflavivirus infection [29] during the antibody response to the whole viral particle (ZIKV), representing mainly cross-reactive epitopes, and leading to ZIKV seroneutralization. The data were used to model the responses to define the kinetic characteristics for comparison between groups of patients. We carried out a statistical comparison of these values to investigate whether there were any correlations between the presence of specific or cross-reactive Abs and their neutralizing function in viral protection.

## Materials and methods

### Ethical approval

Ethical approval was given by the Comité de Protection des Personnes Sud Méditerranée I for the “Etude descriptive prospective de la maladie à virus Zika au sein de la communauté de défense des Forces Armées en Guyane ZIFAG” and was registered on February 29, 2016, as RCB: 2016-A00394-47. All necessary patient informed written consent was obtained, and the appropriate institutional forms were archived. All research was carried out in accordance with relevant guidelines from the Declaration of Helsinki.

### Clinical samples

A one-year prospective cohort study of 49 ZIKV-infected patients was conducted in French Guiana during the 2016 ZIKV outbreak [30]. Clinical data for these patients can be found in previous publications on this cohort [30,31]. ZIKV infection was confirmed by RT-PCR in serum or urine. A sufficient number of samples and an adequate time distribution for each patient are required to model a curve using Wood’s equation. No solution to the Wood equation was found for 16 patients and they were excluded from the study. Serum samples (n = 285) from the 33 remaining patients were collected 7–11 times over one year after symptom onset: on days 3, 5, 7, 14, 21, and 28 and on months 2, 3, 6, 9, and 12. The selected patients were divided into two groups. One contained patients with no previous orthoflavivirus infection, i.e., the earliest sample (day < 4) did not contain IgG against orthoflaviviruses (n = 24; 75% male, 25% female; median age = 39). None of the serum samples from patients without prior DENV infection were able to neutralize ZIKV or DENV before day 4 of the ZIKV infection. The other group comprised patients with a prior DENV infection (n = 9; 56% male, 44% female; median age = 41), i.e., the earliest sample contained IgG against orthoflaviviruses and showed positive seroneutralization for at least one DENV serotype (S1 Table).

### Production and purification of antigens and viruses

The coding sequences of the envelope protein domain III (EDIII) [27] for ZIKV (Polynésie Française, 2013, Accession number KJ776791), DENV1 (DENV1_CNR-SN_VCT_2012, accession number PP695350), DENV2 (Martinique DENV2 98–703 strain 1998, accession number AF208496), DENV3 (Martinique DENV3, accession number AH011666), and DENV4 (DENV4_CNR-SN-IRBA-814_IDN_2014, accession number PP695349) were optimized for production in *E. coli* by GenScript, which included the addition of a His-tag coding sequence to their 5’ ends. These synthesized sequences were then cloned into plasmids pET-24a or pET-19b (Novagen). The recombinant proteins were then produced in *E. coli* T7 Iq Express (New England Biolabs), purified under denaturing conditions, and subjected to in-vitro folding, followed by size-exclusion chromatography for purification, polishing, and assessment of protein homogeneity [32]. The purity of the recombinant proteins was finally verified on SDS PAGE gels stained with Coomassie blue.

Vero cells were exposed to ZIKV or DENV1–4 (accession numbers and strains are the same as those of the EDIII sequences) at a multiplicity of infection of 0.01 and cultured in Dulbecco’s modified Eagle’s medium (DMEM) supplemented with 2% heat-inactivated fetal calf serum (FCS) at 37°C and 5% CO_2_ for 3.5 days (ZIKV) or seven days (DENV), depending on the virus. After incubation, culture supernatants were collected, and viral particles precipitated using polyethylene glycol 6000 (10% w/v PEG 6000) and 1 M NaCl. The resulting precipitates were washed, resuspended in PBS (phosphate buffered saline) supplemented with 7.5 mM HEPES (pH8) solution and, for viruses used as ELISA targets, subjected to viral inactivation with beta-propiolactone.

### Immunoassays (or ELISA)

Briefly, in-house ELISAs were performed to determine IgG and IgM responses to ZIKV and the IgG response to ZEDIII and dengue virus EDIII (DEDIII) from the four serotypes, as described in Denis et al. 2019 [27], to obtain the ODr (OD_(target)_/OD_(noise)_) for each serum sample. Positive thresholds for each antigen were determined by averaging 50 ODr values obtained from a panel of orthoflavivirus-negative sera, adding three standard deviations. The cut-off value for the MAC-ELISA targeting IgG and IgM against Zika virus was set to an ODr = 3. For ZEDIII and DEDIII (1–4), the corresponding values were 1.54, 1.92, 2.09, 1.86, and 1.82, respectively.

### Virus neutralization test

A microneutralization assay was performed to titer the neutralizing Abs from the serum samples in duplicates. After filtration through a 0.22 µm filter, 100 µL of test sera were diluted in PBS and two-fold serial dilutions from 1:20–1:320 were prepared in a 96-well plate. For each dilution, contact with virus was performed using a 50 median tissue culture infectious dose (TCID50) for 1 h at 37°C in a 5% CO_2_ incubator. The complexes were then added to Vero cells (American Tissue Culture Collection (ATCC CCL-81, 1.3 × 10^5^ cells/well). After four days of incubation for ZIKV and seven days for DENV, cytopathic effects were studied under a light microscope by a qualified operator under biosafety level 3 conditions. The neutralizing titer was defined as the inverse of the highest dilution resulting in a 50% reduction in infection. Samples were considered positive if the titer was ≥ 1:40.

### Determination of the kinetic characteristics of the humoral immune response

The kinetic characteristics, such as “day_max_”, referring to the day with the highest amplitude (ODr or neutralizing titer), represented by “level_max_”, were determined by extrapolation by plotting a curve fit to the experimental data using the Wood equation [33, 34].

The adapted Wood equation (ODr (or titer) = a.Day^b^.exp^(-c.Day)^ + d, where d = 1 for ODr) was used to determine the Wood parameters a, b, and c (presented for each patient in S2 Table) after curve fitting using KaleidaGraph 4.5 software. The Level_max_ and day_max_ were calculated using the following equations: level_max_ = a(b/c)^b^exp^(-b)^ and day_max_ = b/c. According to these equations, the calculated titer was determined for each day from day 1 to day 1000.

### Statistical analysis

As most groups did not follow a normal distribution (Shapiro-Wilk test), non-parametric statistical analysis was performed to assess the differences between groups (Kruskal-Wallis test). In the event of a difference between groups, a post-hoc Dunn’s multiple comparison test was used to compare the day_max_ data with the day_max_ of another variable (GraphPad Prism, v10.2 software). To compare the levels for patients with or without an anamnestic response, we used the Mann-Whitney test to check whether the distribution of the two data groups was similar or not. We used Spearman’s correlation test (n, r, p value) to construct a correlation matrix (R studio-2023.12.1-402 software; GraphPad Prism, v10.2 software). Responses on day 28 were compared using a Wilcoxon rank-sum test (GraphPad Prism, v10.2 software).

## Results

### Determination of the kinetic characteristics of the humoral immune response

**Fig 1:**
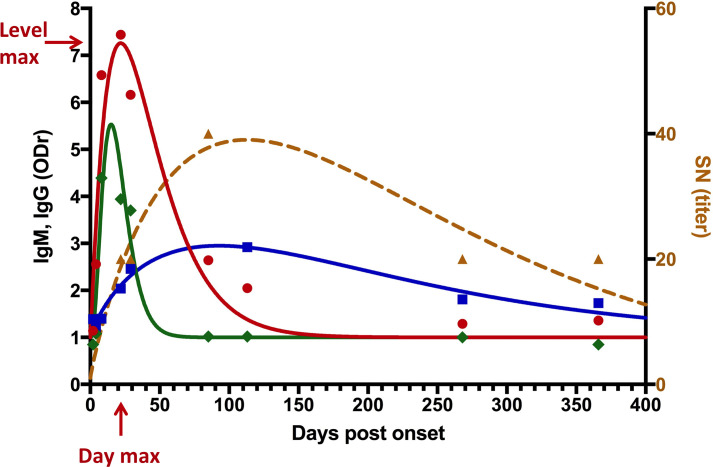
Example of extrapolated kinetic parameters of the immune response for each individual (here Z045): Day_max_ (day with the highest extrapolated immune response) and Level_max_ (highest extrapolated signal of the immune response) for IgM (ZIKV, green), IgG (ZIKV or ZEDIII, red and blue respectively), and seroneutralization (brown).

To compare the immune responses, we defined the characteristics of the kinetics: day_max_, when the immune response was maximal, and level_max_, the signal of the immune response at day_max_. It was possible to recover these characteristics by applying the Wood equation to each immune response using data collected over one year [30]. The model was applied to four different components of the immune response: anti-ZIKV IgM (ZIKV-IgM), anti-ZIKV IgG (ZIKV-IgG), specific anti-ZEDIII IgG (ZEDIII-IgG), and ZIKV neutralizing antibodies (ZIKV-SN) (Figs 1 and S1). For each individual, a dataset for day_max_ and level_max_ were thus available (S2 Table).

### The characteristics of the ZEDIII-specific and whole ZIKV IgG kinetics for the whole cohort are significantly different

**Fig 2:**
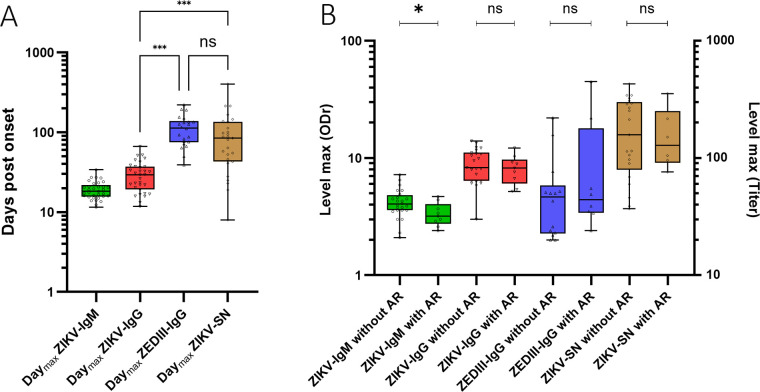
Characteristics of the immune response after Zika virus infection with and without prior DENV infection. **A)** Day_max_ of each immune response determined for ZIKV-IgM, ZIKV-IgG, ZEDIII-IgG, and ZIKV-SN. Groups were compared by one-way ANOVA on ranks (Kruskal-Wallis) with Dunn’s correction (* < 0.05, ** < 0.01, *** < 0.001). The median is represented by the line in the box, the box represents the 25th and 75th percentiles and the error bars represent the minimum and maximum values. **B)** Titer of the immune response at Day_max_ for patients with or without a prior DENV infection for IgM (ZIKV), IgG (ZIKV), IgG (ZEDIII), and ZIKV seroneutralization. Patients with a prior DENV infection are indicated by “AR” (anamnestic response). Immunoglobulin levels refer to the ordinate axis of the ODr (left) and seroneutralization levels to the ordinate axis of the titers (right). Groups were compared by one-way ANOVA on ranks (Kruskal-Wallis) with Dunn’s correction (* < 0.05, ** < 0.01, *** < 0.001). The median is represented by the line in the box, the box represents the 25th and 75th percentiles, and the error bars represent the minimum and maximum values.

The median day_max_ of the whole cohort for ZIKV-IgM (n = 33) was 18.3 days. For ZIKV-IgG (n = 32) and ZEDIII-IgG (n = 22), the medians for the day_max_ were 30 and 114 days, respectively. The Kruskal-Wallis test and a post-hoc Dunn’s multiple comparison showed the day_max_ of ZIKV-IgG to be statistically different from that of ZEDIII-IgG (p = 0.0003). The day_max_ for ZIKV-SN (n = 27) was 85 days (S3 Table). There was a significant difference between the ZIKV-IgG day_max_ and ZIKV-SN day_max_ (p = 0.02), whereas there was no significant difference between the ZEDIII-IgG day_max_ and ZIKV-SN day_max_ (p > 0.99) (Fig 2A). We did not observe any differences in the day_max_ between the values obtained from patients with past infection and those without. These results indicate that ZIKV-IgG peaks significantly earlier than ZEDIII-IgG and ZIKV-SN, suggesting a retarded but sustained EDIII-targeted and neutralizing antibody response. In addition, the similar kinetics of ZEDIII-IgG and ZIKV-SN support the role of EDIII-specific antibodies in viral neutralization.

### Prior dengue infection has an impact on the level of the IgM response

Using the Mann-Whitney test, we found no significant differences in the day_max_ between individuals who were or were not previously infected with DENV for ZIKV-IgM (p = 0.17), ZIKV-IgG (p = 0.47), ZEDIII-IgG (p = 0.41), or ZIKV-SN (p = 0.87) (S2 Fig). However, using the same test, the level_max_ was higher for ZIKV-IgM for patients without an anamnestic response (p = 0.04). There was no difference in the level_max_ (Table 1) between any of the other components of the two groups (Fig 2B). These findings suggest that prior DENV infection does not significantly impact the timing of the antibody response to ZIKV but may influence the magnitude of the IgM response, with higher ZIKV-IgM levels observed in patients without an anamnestic response.

**Table 1:** Level_max_ values obtained following mathematical extrapolation of each patient’s parameters of the whole cohort, without a prior DENV infection, or with a previous DENV infection.

	All patients				Patients with anamnestic response				Patients without anamnestic response			
	Level max	Level max	Level max	Level max	Level max	Level max	Level max	Level max	Level max	Level max	Level max	Level max
	ZIKV-IgM	ZIKV-IgG	ZEDIII-IgG	ZIKV-SN	ZIKV-IgM	ZIKV-IgG	ZEDIII-IgG	ZIKV-SN	ZIKV-IgM	ZIKV-IgG	ZEDIII-IgG	ZIKV-SN
Number of values	33	32	22	27	9	9	8	6	24	23	14	21
												
Minimum	2	3	2	37	2	5	2	76	2	3	2	37
5% Percentile	2	4	2	38	2	5	2	76	2	4	2	37
Median	4	8	5	151	3	8	4	128	4	8	5	158
95% Percentile	7	14	42	401	5	12	45	356	7	14	22	422
Maximum	7	14	45	431	5	12	45	356	7	14	22	431
Range	5	11	43	394	2	7	43	280	5	11	20	394

### ZIKV seroneutralization for the whole cohort is driven by ZEDIII-specific IgG but not whole ZIKV IgG

**Fig 3:**
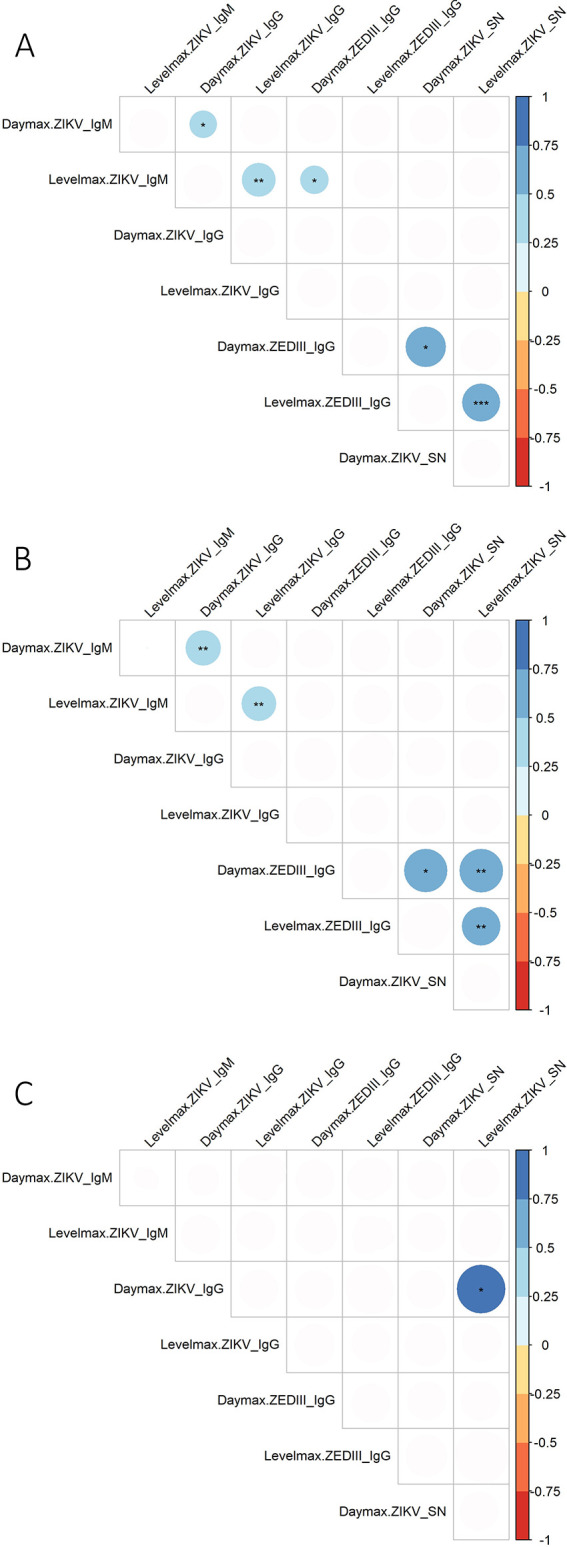
Spearman correlation matrices of the immune response for level_max_ and day_max_ for A) the whole cohort, B) the group of patients without a orthoflavivirus anamnestic response, and C) the group of patients with a orthoflavivirus anamnestic response. The color of the circle represents the value of Spearman’s r on a scale of 1 (blue, positive correlation) to -1 (red, negative correlation). Only statistically significant values are shown (* < 0.05, ** < 0.01, *** < 0.001).

We assessed correlations between the day_max_ and level_max_ of ZIKV-IgM, ZIKV-IgG, ZEDIII-IgG, and ZIKV-SN in the whole cohort using Spearman’s correlation test (Fig 3A and S4A Table). For the whole cohort, there was a significant positive correlation between the ZEDIII-IgG day_max_ and ZIKV-SN day_max_ (n = 18, r = 0.56, p < 0.01), whereas there was no correlation between the ZIKV-IgG day_max_ and ZIKV-SN day_max_ (n = 26, r = 0.06, p = 0.78) or between the ZIKV-IgG day_max_ and ZEDIII-IgG day_max_ (n = 22, r = -0.33, p = 0.14). There was a significant positive correlation between the level_max_ of ZIKV-IgM and the level_max_ of ZIKV-IgG (n = 32, r = 0.49, p < 0.01) and between the level_max_ of ZEDIII-IgG and the level_max_ max of ZIKV-SN (n = 18, r = 0.81, p < 0.01). We also observed another trend between the day_max_ of ZIKV-IgG and the day_max_ of ZIKV-IgM (n = 32, r = 0.43, p < 0.01) (Fig 3A).

### Seroneutralization of ZIKV by sera from patients not previously infected with orthoflavivirus is mainly due to specific antibodies

We compared the immune response and seroneutralization values using Spearman’s correlation test. The results were the following for patients who had not been previously infected (Fig 3B and S4B Table). The medians of the day_max_ for ZIKV-IgM (n = 24), ZIKV-IgG (n = 23), ZEDIII-IgG (n = 14), and ZIKV-SN (n = 21) were 19, 32, 119, and 85 days, respectively (S3 Table). There was a significant positive correlation between the ZEDIII-IgG day_max_ and ZIKV-SN day_max_ (n = 13, r = 0.6, p = 0.04), whereas there was no correlation between the ZIKV-IgG day_max_ and the ZIKV-SN day_max_ (n = 21, r = 0.09, p = 0.71) or between the ZIKV-IgG day_max_ and ZEDIII-IgG day_max_ (n = 14, r = -0.39, p = 0.17). We assessed the statistical trends between the ZIKV-IgG day_max_ and ZIKV-IgM day_max_ (n = 23, r = 0.56, p = 0.01) and between the ZIKV-IgM level_max_ and ZIKV-IgG level_max_ (n = 23, r = 0.55, p < 0.01). There was a significant correlation between the ZEDIII-IgG level_max_ and ZIKV-SN level_max_ (n = 18, r = 0.80, p < 0.01).

### Past infection with dengue virus promotes the production of cross-reactive seroneutralizing IgG against infecting ZIKV and hides the detection of specific seroneutralization by ZIKV-IgG

The results for patients with prior DENV infection were as follows. The medians of the day_max_ for ZIKV-IgM (n = 9), ZIKV-IgG (n = 9), ZEDIII-IgG (n = 8), and ZIKV-SN (n = 6) were 16, 24, 96, and 78 days, respectively (S3 Table). There was a correlation between the ZIKV-SN level_max_ and ZIKV-IgG day_max_ (n = 6, r = 0.89, p = 0.03) (Figs 3C, S2 and S4C Table). The significant positive correlation between the ZEDIII-IgG day_max_ and ZIKV-SN day_max_ found in patients without prior DENV infection was not found in the previously infected patients (n = 5, r = 0.5, p = 0.45), nor that between ZEDIII-IgG level_max_ and ZIKV-SN level_max_ (n = 5, r = 0.00, p = 0.08).

### DENV-independent seroneutralization of anti DEDIII-IgG

**Fig 4:**
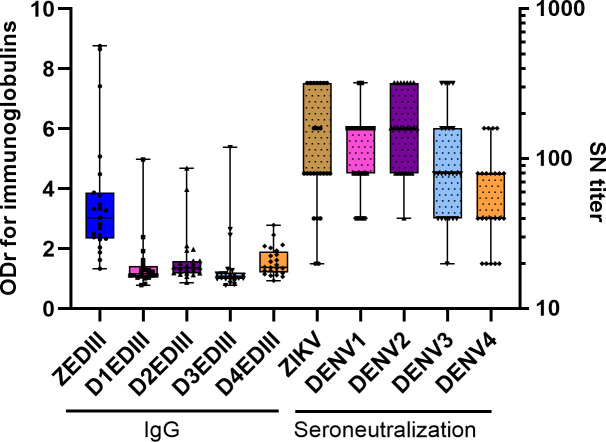
Cross-reaction and cross-neutralization on 28 days post-onset of symptoms. Box plot (Min-Max) of the optical density ratio values for IgM and IgG DIII and serum neutralization titers for DENV and ZIKV 28 days post-onset of symptoms for patients without prior DENV infection. Immunoglobulin levels (non-dotted) refer to the ordinate axis of the ODr (left) and seroneutralization levels (dotted boxes) to the ordinate axis of the titers (right).

We assessed EDIII-IgG levels and seroneutralization titers for the four DENV serotypes and ZIKV from sera collected on day 28 for patients without an anamnestic response. Using predefined positivity thresholds, we calculated the percentage of positive sera across groups (Table 2). IgG positivity was 96% for ZEDIII and ranged from 9% to 25% for DENV-EDIII, while seroneutralization was ≥ 92% for ZIKV and DENV1–3 and lower for DENV4 (78%) (Fig 4). For patients with an anamnestic response, IgG positivity remained 100% for ZEDIII but varied more for DENV (11% - 44%). A comparison of the IgG and SN responses for patients without an anamnestic response using the Wilcoxon test showed no significant difference between ZIKV-SN and ZEDIII-IgG (p > 0.9999). However, there were significant differences for all four DENV serotypes (p < 0.0001 for DENV1, DENV2, and DENV3; p = 0.0013 for DENV4). Finally, the Kruskal-Wallis test and a post-hoc Dunn’s multiple comparison showed no significant difference in seroneutralization between patients with and without prior DENV infection for any DENV serotype (p > 0.99 for DENV1, DENV2, and DENV4; p = 0.1672 for DENV3). This highlights the presence of DENV seroneutralization in the absence of DENV-EDIII IgG, suggesting the presence of orthoflavivirus-specific (but not ZIKV-specific) IgG in the short term, at day 28.

**Table 2:** Percentage of positive EDIII IgG and seroneutralization sera to ZIKV or DENV serotypes. AR: anamnestic response (prior DENV infection).

	IgG ZEDIII	IgG D1EDIII	IgG D2EDIII	IgG D3EDIII	IgG D4EDIII	SN ZIKV	SN DENV1	SN DENV2	SN DENV3	SN DENV4
Day 28 (whole cohort)	94	10	12	13	29	91	100	100	97	72
Day 28 (without AR)	96	9	13	14	25	92	100	100	96	78
Day 28 (with AR)	100	13	22	11	44	88	100	100	100	63

## Discussion

The dynamics of the humoral response to ZIKV infection has already been described [35]. In particular, in a cohort of children in Nicaragua, Montoya et al. showed that ZIKV infection elicits cross-neutralizing antibodies to DENV with a similar titer to that of anti-ZIKV antibodies, independently of prior DENV infection [36]. The magnitude of the antibody response to ZIKV is only modestly influenced by immunity induced by prior DENV infection [16]. However, higher levels of ZIKV-neutralizing antibodies have been observed in DENV-immune donors than DENV-naïve donors [37]. Here, we modeled the immune response induced by ZIKV infection to extrapolate values that allow for meaningful comparisons. The outcome of an infection - from asymptomatic to severe with severe clinical complications - depends on the speed with which the immune response is established. The dynamics of viral multiplication and the kinetics of the immune response thus play an important role. In this study, and for the first time, we determined several parameters of the kinetics associated with the immune response of 33 symptomatic patients. This approach to individual kinetics could provide essential information for vaccine efficacy studies in areas where several orthoflavivirus are circulating, as an increasing number of vaccines are brought to market.

Cross-neutralizing antibodies from DENV-exposed individuals can neutralize ZIKV [38], and we demonstrate that prior DENV infection influences the type of antibodies, specific or cross-neutralizing, involved in ZIKV neutralization. We characterized the kinetics of antibody production and seroneutralization in a cohort of 33 patients, either naïve or previously infected with DENV. We were able to determine the day on which the responses were maximal, as well as the maximum amplitude. We considered IgG directed against ZEDIII to be a specific response directed against ZIKV [22,27]. A previous study showed that cross-reacting antibodies target ZEDIII in the early convalescent phase (2–12 weeks post-infection) of secondary DENV infections [29]. The antibody levels were characterized by quantifying ZEDIII-specific IgG titers. In our study, ZEDIII IgG levels were assessed using the ODr, allowing the detection of both high- and low-affinity IgG. This approach differs from IgG titer measurement, which exclusively reflects the concentration of high-affinity IgG targeting their antigen. ZEDIII has been identified as a neutralizing epitope, but other specific epitopes are present on the viral surface [39]. Specific antibodies targeting quaternary epitopes, such as the interface between the two E subunits of the dimer, have been shown to neutralize both DENV and ZIKV [40]. By contrast, we considered that IgG directed against the whole virus, carrying mostly orthoflavivirus-specific epitopes, were mainly representative of cross-reactivity, as previously shown [27,41], although certain epitopes are specifically recognized [28]. Comparison of the kinetics of virus-specific and orthoflavivirus cross-reactive responses, stratified by DENV infection history, allowed us to identify the antibody types likely involved in ZIKV and DENV seroneutralization. ZIKV seroneutralization by sera from ZIKV-infected individuals appeared to be mainly attributable to virus-specific Abs. However, our findings indicate that sera from the same individuals also exhibited the ability to seroneutralize all four DENV serotypes in a non-ZIKV-specific manner. In individuals with prior DENV infection, ZIKV seroneutralization appeared to be predominantly mediated by cross-reactive antibodies. As anticipated, these cross-reactive antibodies also demonstrated seroneutralizing activity against all four DENV serotypes. Anamnestic responses to DENV may have hindered the detection of de-novo ZIKV-specific antibodies. This anamnestic response involved non-neutralizing Abs that may contribute to antibody-dependent enhancement (ADE), a phenomenon involved in secondary infection [42,43].

The tight association observed between EDIII-specific Abs and seroneutralization was to be expected, as this domain of the E protein is the site of attachment of the virus to the host cell. Two arguments reinforce our conclusions. First, other specific-neutralizing epitopes are present on the surface of the two viruses, but there was no correlation between this specific response and ZIKV seroneutralization in patients without prior DENV infection. Second, this correlation was strongly observed in the absence of a second infection. The absence of anti-DENV-EDIII antibody detection in conjunction with seroneutralization is the most convincing evidence of the robustness of our results.

Our test results showed that there was no correlation between IgM and serum neutralization, contrary to the results of previous studies [44]. However, as observed on day 28, it is also possible that the presence of IgM (79% positive) may have had a small impact on seroneutralization during the acute phase. Nevertheless, the amplitude of the IgM response was found to be lower in cases of a second infection. This difference has already been reported in the literature for second DENV infections [45,46], thus reinforcing the reliability of our model. A decrease in the involvement of specific antibodies during the second infection was also expected, with the observation of a reduction in the de-novo IgM response, which should be more specific.

The increased involvement of cross-reactive responses in viral seroneutralization during sequential infection has previously been described in the literature [47,48]. However, the involvement of IgG responses in the seroneutralization of the infecting virus and that of homologous viruses, as in this study, had not been described previously. Here, we were able to delineate the respective contributions of virus-specific and orthoflavivirus-cross-reactive antibodies to the seroneutralization of both infecting and antigenically related viruses. It appears that antibodies targeting DENV/ZIKV and the yellow fever virus (YFV) do not cross-react due to genetic differences. One of the limitations of this study was the lack of biological material available to assess the duration of seroneutralization and that of other approaches such as antibody purification or depletion. It would have been informative to examine the impact of both primary and secondary infections on the efficacy of YFV vaccination, providing insights into the anamnestic response associated with immunization against various orthoflaviviruses.

Serum neutralization remains the gold standard for detecting specific immunoglobulins and identifying the infecting virus in many reference laboratories. However, our findings indicate that following ZIKV infection, selected antibodies not only neutralize ZIKV but also all four DENV serotypes. This highlights the limitations of standard seroneutralization assays in distinguishing between closely related orthoflaviviruses. In our study, ELISAs targeting EDIII appeared to be the most reliable approach, with the presence of IgM antibodies against this domain emerging as a particularly strong signal. Nonetheless, serological results alone cannot replace genome detection, which remains the only method recognized by the WHO for definitive case confirmation.

Seroneutralization with sera from patients who had not been previously infected with DENV is less durable than the ZIKV-specific immune response [19,49]. Consequently, seroneutralization tests conducted shortly after infection (usually asymptomatic) in orthoflavivirus-endemic regions offer an unreliable assessment of the infecting virus and the long-term protection a patient can expect. This underlines the need for serological tests specifically targeting unique viral markers to ensure diagnostic reliability. The observation of transient but enhanced cross-neutralization following heterologous orthoflavivirus infection also raises important considerations for clinical management and vaccination strategies. Specifically, it highlights the potential implications of sequential exposures to orthoflaviviruses - whether natural or vaccine-induced - on long-term humoral immunity. Understanding the dynamics and specificity of cross-reactive antibody responses may help guide the design of multivalent vaccines that simultaneously target multiple orthoflaviviruses, as suggested by recent studies exploring pan-orthoflavivirus vaccine strategies [50]. Future studies are needed to determine whether such exposure enhances vaccine-induced immunity by reactivating cross-reactive memory B cells, and whether repeated stimulation contributes to longer-lasting or broader protection. Thus, it may be relevant for vaccine developers to assess the impact of candidate vaccines on related orthoflaviviruses during late-stage clinical trials, particularly in endemic areas.

## Supporting information

S1 Table Description of all patients together or separated into 2 groups: with and without a flavivirus anamnestic response. (DOCX)

S1 Fig Kinetics of immune responses for all individualized patients. The kinetics of IgM-ZIKV (green), IgG-ZIKV (red), IgG-ZEDIII (blue), and SN-ZIKV (brown) were extrapolated from experimental values from samples using the Wood equation. The right axis indicates the amplitude of immunoglobulins and the left axis the amplitude of seroneutralization.
(DOCX)

S2 Fig Day_max_ of each immune response for patients with or without a previous dengue infection (AR: anamnestic response). (DOCX)

S2 Table Individual parameters from Wood’s equation and hidden data for all patients. (DOCX)

S3 Table Distribution of the Day_max_ of all patients, patients with an anamnestic response, and patients without an anamnestic response. (DOCX)

S4 Table Spearman correlation matrix for patients: A) the whole cohort, B) patients without a flavivirus anamnestic response, and C) patients with a flavivirus anamnestic response. The Spearman r values are shown to the right of the diagonal and the p-value and number of individuals to the left. Bold underlined r values are those with a significant p-value highlighted in green.
(DOCX)

S5 Table A) Patients on day 28 without a previous DENV infection B) Patients on day 28 with a previous DENV infection. The positive threshold ODr for IgM and IgG against ZIKV were 3. The positive threshold ODrs for IgG to ZEDIII, D1EDIII, D2EDIII, D3EDIII, and D4EDIII were 1.54, 1.92, 1.52, 1.86, and 1.89, respectively. The positive threshold titer of SN was 1/40.
(DOCX)
