# Supplementary material for: Zika virus-specific and orthoflavivirus-cross-reactive IgGs correlate with Zika virus seroneutralization depending on prior dengue virus infection
Source: PLoS Negl Trop Dis. 2025 Jul 9;19(7):e0013274. doi: 10.1371/journal.pntd.0013274 (PMC12240325; doi:10.1371/journal.pntd.0013274)
Supplement: S1 Table — (DOCX) [file pntd.0013274.s001.docx]

| Whole cohort | Groups |  |
| --- | --- | --- |
|  | Number, n | 33 |
|  | Gender, n women (%), n men (%) | 10 (32%), 23 (68%) |
|  | Age in years, median, IQR (range) | 40, 34-45, (27-64) |
|  |  |  |
| Without anamnestic response | Groups |  |
|  | Number, n | 24 |
|  | Gender, n women (%), n men (%) | 6 (25%), 18 (75%) |
|  | Age in years, median, IQR (range) | 39, 32-45, (27-64) |
|  |  |  |
| With anamnestic response | Groups |  |
|  | Number, n | 9 |
|  | Gender, n women (%), n men (%) | 4 (44%), 5 (56%) |
|  | Age in years, median, IQR (range) | 41, 37-44, (21-60) |

Supplementary Table 1. Description of all patients or separated into 2 groups: with and without a flavivirus anamnestic response
