# Supplementary material for: Zika virus-specific and orthoflavivirus-cross-reactive IgGs correlate with Zika virus seroneutralization depending on prior dengue virus infection
Source: PLoS Negl Trop Dis. 2025 Jul 9;19(7):e0013274. doi: 10.1371/journal.pntd.0013274 (PMC12240325; doi:10.1371/journal.pntd.0013274)
Supplement: S1 Fig — The kinetics of IgM-ZIKV (green), IgG-ZIKV (red), IgG-ZEDIII (blue), and SN-ZIKV (brown) were extrapolated from experimental values from samples using the Wood equation. The right axis indicates the amplitude of immunoglobulins and the left axis the amplitude of seroneutralization. (DOCX) [file pntd.0013274.s002.docx]

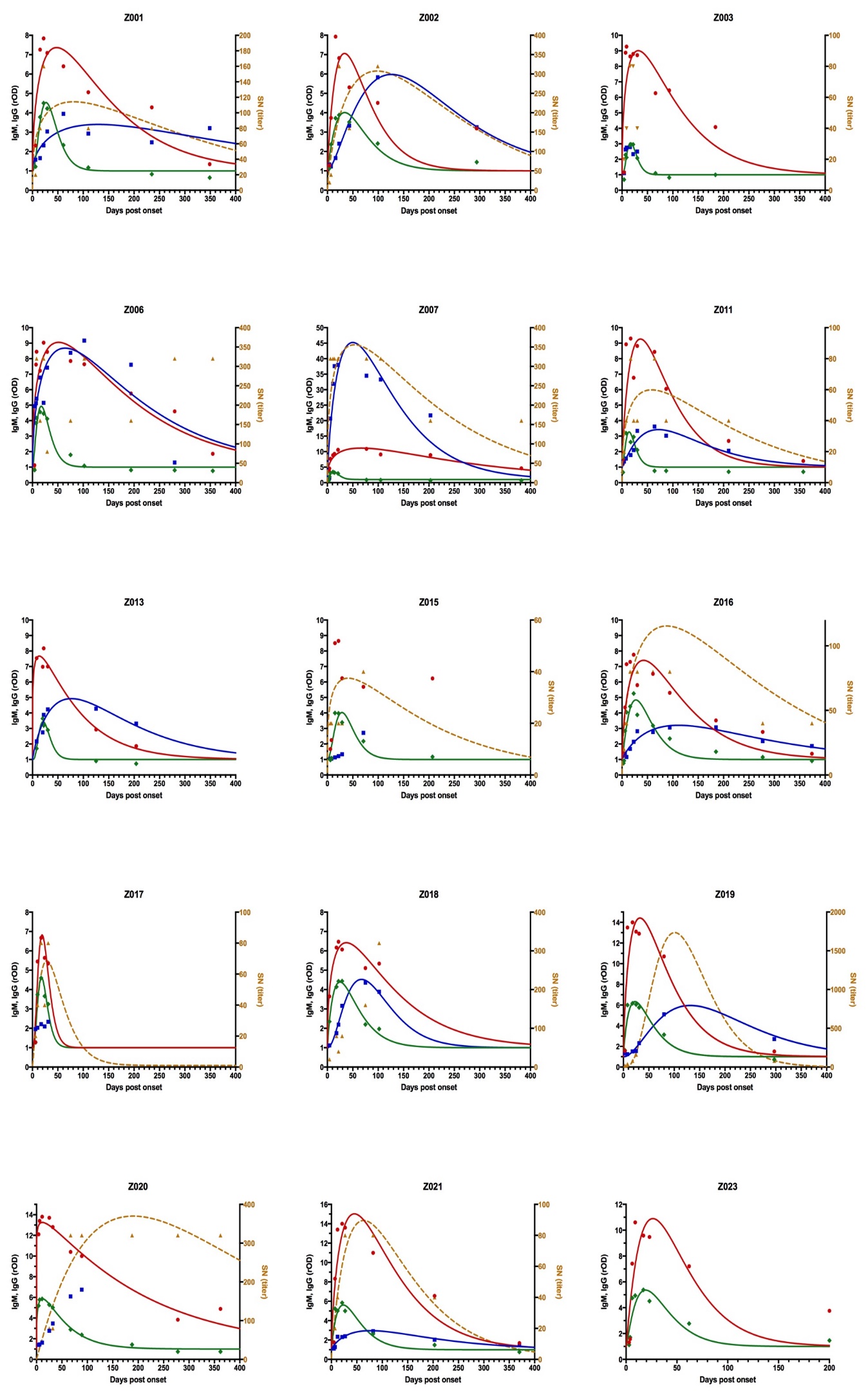


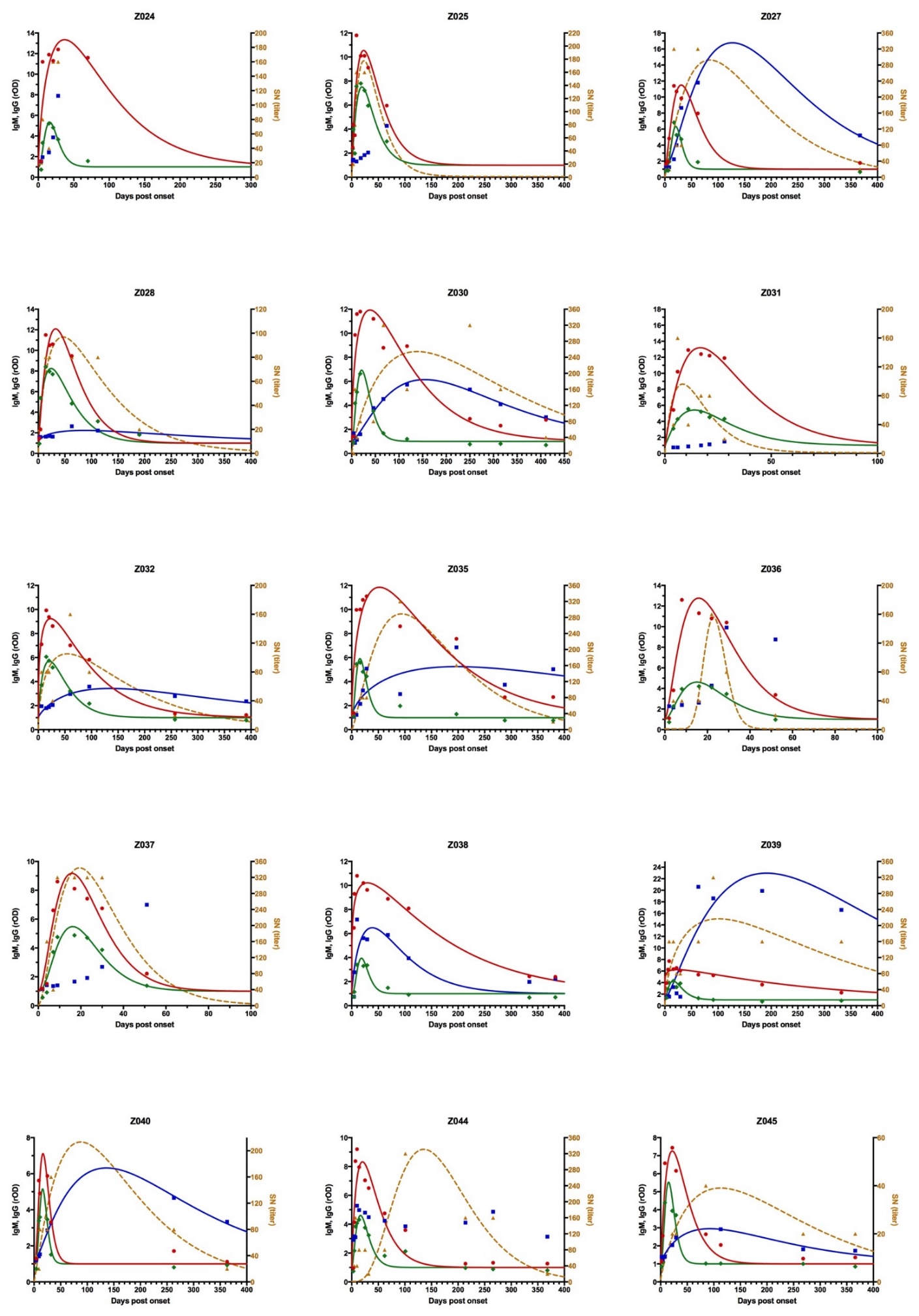


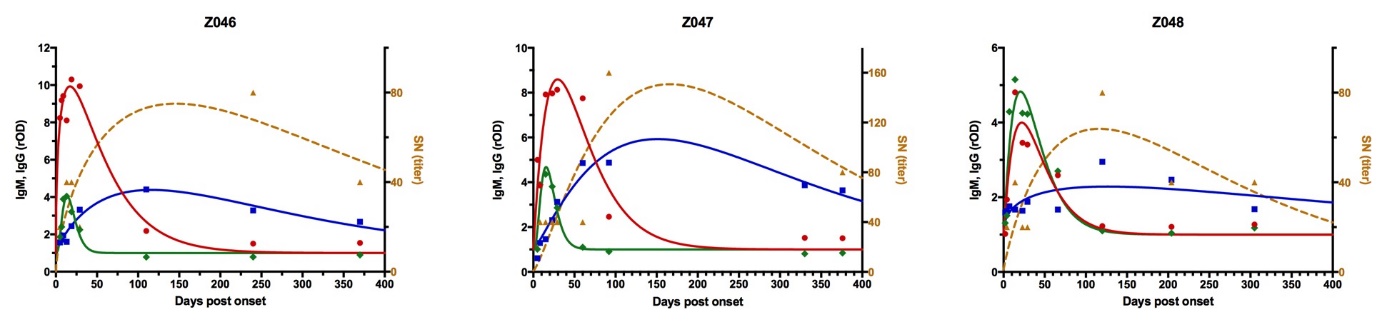


Supplementary Figure 1. Kinetics of immune responses for all individualized patients

The kinetics of IgM-ZIKV (green), IgG-ZIKV (red), IgG-ZEDIII (blue), and SN-ZIKV (brown) were extrapolated from experimental values from samples using the Wood equation. The right axis indicates the amplitude of immunoglobulins and the left axis the amplitude of seroneutralization.
