# Supplementary material for: Zika virus-specific and orthoflavivirus-cross-reactive IgGs correlate with Zika virus seroneutralization depending on prior dengue virus infection
Source: PLoS Negl Trop Dis. 2025 Jul 9;19(7):e0013274. doi: 10.1371/journal.pntd.0013274 (PMC12240325; doi:10.1371/journal.pntd.0013274)
Supplement: S2 Fig — (DOCX) [file pntd.0013274.s003.docx]

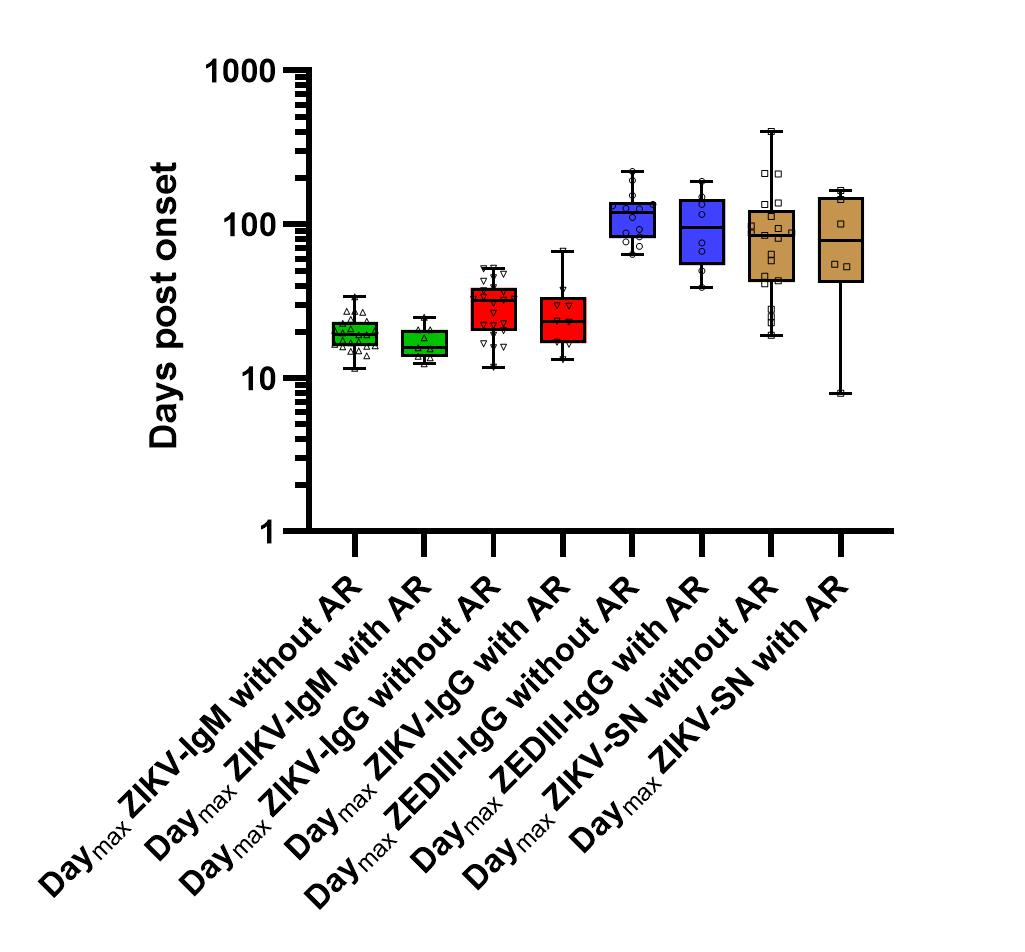


Supplementary Figure 2. Day_max_ of each immune response for patients with or without a previous dengue infection (AR: Anamnestic Response)
