# Supplementary material for: Zika virus-specific and orthoflavivirus-cross-reactive IgGs correlate with Zika virus seroneutralization depending on prior dengue virus infection
Source: PLoS Negl Trop Dis. 2025 Jul 9;19(7):e0013274. doi: 10.1371/journal.pntd.0013274 (PMC12240325; doi:10.1371/journal.pntd.0013274)
Supplement: S2 Table — (DOCX) [file pntd.0013274.s004.docx]

| **#** | **Gender** | **age** | **immune Scare** | **data** | **R^2^** | **a** | **b** | **c** | **Day**_max_ | **Level**_max_ |
| --- | --- | --- | --- | --- | --- | --- | --- | --- | --- | --- |
| Z001 | M | 31 | 0 | IgM ZIKV | 0.97174 | 0.014597 | 2.3991 | 0.089609 | 27 | 3.5 |
|  |  |  |  | IgG ZIKV | 0.72414 | 1.3555 | 0.54064 | 0.011374 | 48 | 6.4 |
|  |  |  |  | IgG ZEDIII | 0.78449 | 0.092566 | 0.97392 | 0.011703 | 83 | 2.6 |
|  |  |  |  | SN ZIKV | 0.21342 | 35.66 | 0.34267 | 0.0042162 | 81 | 114.2 |
| Z002 | M | 53 | 0 | IgM ZIKV | 0.87165 | 0.17562 | 1.1255 | 0.033167 | 34 | 3.0 |
|  |  |  |  | IgG ZIKV | 0.52547 | 0.57225 | 0.93823 | 0.027908 | 34 | 6.1 |
|  |  |  |  | IgG ZEDIII | 0.99058 | 0.0099388 | 1.6195 | 0.012829 | 126 | 5.0 |
|  |  |  |  | SN ZIKV | 0.63673 | 22.158 | 0.7345 | 0.0075187 | 98 | 307.7 |
| Z003 | M | 40 | 0 | IgM ZIKV | 0.90889 | 0.0068978 | 3.1114 | 0.18322 | 17 | 2.1 |
|  |  |  |  | IgG ZIKV | 0.46972 | 2.4233 | 0.48464 | 0.015143 | 32 | 8.0 |
|  |  |  |  | IgG ZEDIII | 0.62892 | 0.091431 | 1.7582 | 0.11474 | 15 | 1.9 |
|  |  |  |  | SN ZIKV | 0.92329 | 0.11748 | 3.5163 | 0.20142 | 17 | 81.3 |
| Z006 | M | 45 | 0 | IgM ZIKV | 0.89235 | 0.13374 | 1.8257 | 0.10543 | 17 | 3.9 |
|  |  |  |  | IgG ZIKV | 0.63745 | 2.3307 | 0.42156 | 0.0081894 | 51 | 8.1 |
|  |  |  |  | IgG ZEDIII | 0.63658 | 1.4519 | 0.52678 | 0.0082031 | 64 | 7.7 |
|  |  |  |  | SN ZIKV | ND | ND | ND | ND | ND | ND |
| Z007 | F | 59 | 1 | IgM ZIKV | 0.88606 | 0.064535 | 2.4221 | 0.19472 | 12 | 2.6 |
|  |  |  |  | IgG ZIKV | 0.81859 | 3.0317 | 0.37795 | 0.0056665 | 67 | 10.2 |
|  |  |  |  | IgG ZEDIII | 0.71787 | 4.5725 | 0.77914 | 0.015581 | 50 | 44.2 |
|  |  |  |  | SN ZIKV | 0.45495 | 120.06 | 0.36418 | 0.0068382 | 53 | 354.8 |
| Z011 | M | 37 | 0 | IgM ZIKV | 0.86486 | 0.060456 | 2.2096 | 0.15746 | 14 | 2.3 |
|  |  |  |  | IgG ZIKV | 0.75288 | 1.0035 | 0.81372 | 0.022422 | 36 | 8.3 |
|  |  |  |  | IgG ZEDIII | 0.86168 | 0.061804 | 1.1204 | 0.015594 | 72 | 2.4 |
|  |  |  |  | SN ZIKV | 0.28956 | 17.67 | 0.39236 | 0.0067219 | 58 | 58.9 |
| Z013 | F | 31 | 1 | IgM ZIKV | 0.97603 | 0.0017929 | 3.5535 | 0.17156 | 21 | 2.4 |
|  |  |  |  | IgG ZIKV | 0.9759 | 4.9361 | 0.19086 | 0.014482 | 13 | 6.7 |
|  |  |  |  | IgG ZEDIII | 0.82445 | 0.22282 | 0.86004 | 0.011256 | 76 | 3.9 |
|  |  |  |  | SN ZIKV | ND | ND | ND | ND | ND | ND |
| Z015 | F | 28 | 0 | IgM ZIKV | 0.79142 | 0.011894 | 2.3492 | 0.081767 | 29 | 3.0 |
|  |  |  |  | IgG ZIKV | 0.33574 | 1.1467 | 0.53768 | 0.0072521 | 74 | 6.8 |
|  |  |  |  | IgG ZEDIII | ND | ND | ND | ND | ND | ND |
|  |  |  |  | SN ZIKV | 0.029048 | 17.837 | 0.27412 | 0.0067074 | 41 | 37.5 |
| Z016 | M | 42 | 0 | IgM ZIKV | 0.83125 | 0.18778 | 1.3089 | 0.04799 | 27 | 3.8 |
|  |  |  |  | IgG ZIKV | 0.70025 | 1.0494 | 0.65774 | 0.015493 | 42 | 6.4 |
|  |  |  |  | IgG ZEDIII | 0.90872 | 0.081397 | 0.88882 | 0.0079857 | 111 | 2.2 |
|  |  |  |  | SN ZIKV | 0.38572 | 19.186 | 0.51603 | 0.0058657 | 88 | 115.4 |
| Z017 | M | 36 | 0 | IgM ZIKV | 0.86331 | 0.0059759 | 3.4235 | 0.1937 | 18 | 3.6 |
|  |  |  |  | IgG ZIKV | 0.8284 | 0.016414 | 3.0222 | 0.15937 | 19 | 5.8 |
|  |  |  |  | IgG ZEDIII | ND | ND | ND | ND | ND | ND |
|  |  |  |  | SN ZIKV | 0.5324 | 3.1007 | 1.3261 | 0.047225 | 28 | 68.6 |
| Z018 | F | 41 | 1 | IgM ZIKV | 0.97808 | 0.42636 | 0.93703 | 0.037689 | 25 | 3.4 |
|  |  |  |  | IgG ZIKV | 0.83335 | 1.5868 | 0.46856 | 0.012522 | 37 | 5.4 |
|  |  |  |  | IgG ZEDIII | 0.98315 | 0.0024708 | 2.2639 | 0.033662 | 67 | 3.5 |
|  |  |  |  | SN ZIKV | ND | ND | ND | ND | ND | ND |
| Z019 | F | 34 | 0 | IgM ZIKV | 0.91474 | 0.77761 | 0.90384 | 0.039581 | 23 | 5.3 |
|  |  |  |  | IgG ZIKV | 0.84504 | 2.2336 | 0.72224 | 0.022156 | 33 | 13.4 |
|  |  |  |  | IgG ZEDIII | 0.94347 | 0.0018635 | 2.032 | 0.015418 | 132 | 5.0 |
|  |  |  |  | SN ZIKV | 0.95074 | 0.0056405 | 3.4906 | 0.03457 | 101 | 1703.1 |
| Z020 | M | 42 | 0 | IgM ZIKV | 0.99248 | 3.2036 | 0.28713 | 0.024692 | 12 | 4.9 |
|  |  |  |  | IgG ZIKV | 0.95376 | 11.175 | 0.061831 | 0.0052452 | 12 | 12.2 |
|  |  |  |  | IgG ZEDIII | 0.97746 | 0.00066167 | 2.3664 | 0.010669 | 222 | 22.1 |
|  |  |  |  | SN ZIKV | 0.50241 | 17.447 | 0.68263 | 0.0031878 | 214 | 343.8 |
| Z021 | M | 29 | 0 | IgM ZIKV | 0.82924 | 0.59347 | 0.95225 | 0.040689 | 23 | 4.6 |
|  |  |  |  | IgG ZIKV | 0.76172 | 1.8778 | 0.71643 | 0.01589 | 45 | 14.0 |
|  |  |  |  | IgG ZEDIII | 0.8133 | 0.12932 | 0.81286 | 0.010517 | 77 | 2.0 |
|  |  |  |  | SN ZIKV | 0.91915 | 5.3762 | 0.89228 | 0.01403 | 64 | 89.6 |
| Z023 | M | 35 | 0 | IgM ZIKV | 0.82249 | 0.39211 | 1.2311 | 0.064006 | 19 | 4.4 |
|  |  |  |  | IgG ZIKV | 0.7225 | 0.86448 | 1.0729 | 0.040716 | 26 | 9.9 |
|  |  |  |  | IgG ZEDIII | ND | ND | ND | ND | ND | ND |
|  |  |  |  | SN ZIKV | ND | ND | ND | ND | ND | ND |
| Z024 | M | 26 | 0 | IgM ZIKV | 0.88227 | 0.031495 | 2.7773 | 0.17341 | 16 | 4.3 |
|  |  |  |  | IgG ZIKV | 0.57497 | 1.911 | 0.71427 | 0.019275 | 37 | 12.3 |
|  |  |  |  | IgG ZEDIII | ND | ND | ND | ND | ND | ND |
|  |  |  |  | SN ZIKV | ND | ND | ND | ND | ND | ND |
| Z025 | M | 29 | 0 | IgM ZIKV | 0.72162 | 0.59003 | 1.2285 | 0.064002 | 19 | 6.5 |
|  |  |  |  | IgG ZIKV | 0.71461 | 1.2236 | 0.9713 | 0.043199 | 22 | 9.5 |
|  |  |  |  | IgG ZEDIII | ND | ND | ND | ND | ND | ND |
|  |  |  |  | SN ZIKV | 0.81564 | 15.887 | 1.1292 | 0.048973 | 23 | 177.6 |
| Z027 | F | 43 | 0 | IgM ZIKV | 0.91957 | 0.00044593 | 4.6444 | 0.22637 | 21 | 5.3 |
|  |  |  |  | IgG ZIKV | 0.90744 | 0.19598 | 1.6405 | 0.053274 | 31 | 10.5 |
|  |  |  |  | IgG ZEDIII | 0.93324 | 0.032182 | 1.6132 | 0.012727 | 127 | 15.8 |
|  |  |  |  | SN ZIKV | 0.49387 | 6.183 | 1.1218 | 0.01327 | 85 | 292.3 |
| Z028 | M | 55 | 0 | IgM ZIKV | 0.86564 | 1.1584 | 0.83982 | 0.034822 | 24 | 7.2 |
|  |  |  |  | IgG ZIKV | 0.89963 | 0.57923 | 1.189 | 0.036473 | 33 | 11.1 |
|  |  |  |  | IgG ZEDIII | 0.56153 | 0.20618 | 0.51701 | 0.0059622 | 87 | 1.2 |
|  |  |  |  | SN ZIKV | 0.87905 | 11.954 | 0.73778 | 0.01594 | 46 | 96.8 |
| Z030 | M | 39 | 0 | IgM ZIKV | 0.9514 | 0.07623 | 2.133 | 0.10194 | 21 | 5.9 |
|  |  |  |  | IgG ZIKV | 0.73218 | 2.6703 | 0.53073 | 0.013703 | 39 | 10.9 |
|  |  |  |  | IgG ZEDIII | 0.97341 | 0.01493 | 1.4459 | 0.0093553 | 155 | 5.1 |
|  |  |  |  | SN ZIKV | 0.4495 | 7.3668 | 0.90105 | 0.0065193 | 138 | 253.9 |
| Z031 | M | 39 | 1 | IgM ZIKV | 0.89617 | 0.60889 | 1.2158 | 0.087609 | 14 | 4.4 |
|  |  |  |  | IgG ZIKV | 0.86122 | 1.6109 | 1.1175 | 0.067229 | 17 | 12.2 |
|  |  |  |  | IgG ZEDIII | 0.33818 | -0.035859 | 3.029 | 0.57201 | 5 | -0.3 |
|  |  |  |  | SN ZIKV | 0.22572 | 32.796 | 0.95368 | 0.11352 | 8 | 96.2 |
| Z032 | M | 42 | 1 | IgM ZIKV | 0.97558 | 0.84118 | 0.84819 | 0.040963 | 21 | 4.7 |
|  |  |  |  | IgG ZIKV | 0.97541 | 3.7254 | 0.37074 | 0.016049 | 23 | 8.2 |
|  |  |  |  | IgG ZEDIII | 0.84104 | 0.11147 | 0.78885 | 0.0058377 | 135 | 2.4 |
|  |  |  |  | SN ZIKV | 0.49214 | 21.402 | 0.53015 | 0.0097205 | 55 | 104.9 |
| Z035 | M | 46 | 0 | IgM ZIKV | 0.90226 | 0.064436 | 2.49 | 0.16079 | 15 | 4.9 |
|  |  |  |  | IgG ZIKV | 0.73693 | 2.0822 | 0.55786 | 0.010692 | 52 | 10.8 |
|  |  |  |  | IgG ZEDIII | 0.56958 | 0.28069 | 0.63392 | 0.0032127 | 197 | 4.2 |
|  |  |  |  | SN ZIKV | 0.67592 | 2.1078 | 1.3888 | 0.014802 | 94 | 288.3 |
| Z036 | F | 39 | 0 | IgM ZIKV | 0.92841 | 0.12497 | 1.9635 | 0.13021 | 15 | 3.6 |
|  |  |  |  | IgG ZIKV | 0.86528 | 0.68366 | 1.6124 | 0.10153 | 16 | 11.8 |
|  |  |  |  | IgG ZEDIII | ND | ND | ND | ND | ND | ND |
|  |  |  |  | SN ZIKV | 0.71839 | 8.4219E-18 | 20.829 | 0.9101 | 23 | 157.8 |
| Z037 | M | 46 | 0 | IgM ZIKV | 0.89624 | 0.053888 | 2.4799 | 0.15346 | 16 | 4.5 |
|  |  |  |  | IgG ZIKV | 0.88139 | 0.23539 | 2.0166 | 0.12772 | 16 | 8.2 |
|  |  |  |  | IgG ZEDIII | ND | ND | ND | ND | ND | ND |
|  |  |  |  | SN ZIKV | 0.63394 | 10.611 | 1.7703 | 0.09129 | 19 | 343.9 |
| Z038 | M | 47 | 1 | IgM ZIKV | 0.8758 | 0.0087036 | 3.0532 | 0.16677 | 18 | 2.9 |
|  |  |  |  | IgG ZIKV | 0.92306 | 5.3962 | 0.22544 | 0.0076645 | 29 | 9.2 |
|  |  |  |  | IgG ZEDIII | 0.59043 | 0.6422 | 0.80863 | 0.020974 | 39 | 5.5 |
|  |  |  |  | SN ZIKV | ND | ND | ND | ND | ND | ND |
| Z039 | M | 41 | 1 | IgM ZIKV | 0.92529 | 0.22602 | 1.5125 | 0.095646 | 16 | 3.2 |
|  |  |  |  | IgG ZIKV | 0.66618 | 4.1593 | 0.10677 | 0.0045471 | 23 | 5.2 |
|  |  |  |  | IgG ZEDIII | 0.85295 | 0.092568 | 1.2859 | 0.0067244 | 191 | 22.0 |
|  |  |  |  | SN ZIKV | 0.39597 | 25.597 | 0.59014 | 0.0058215 | 101 | 216.6 |
| Z040 | F | 63 | 0 | IgM ZIKV | 0.9402 | 0.00027994 | 5.4015 | 0.33586 | 16 | 4.1 |
|  |  |  |  | IgG ZIKV | 0.77138 | 0.10033 | 2.3332 | 0.14048 | 17 | 6.8 |
|  |  |  |  | IgG ZEDIII | 0.99134 | 0.036768 | 1.2709 | 0.0093315 | 136 | 5.3 |
|  |  |  |  | SN ZIKV | 0.75643 | 3.9636 | 1.1452 | 0.01307 | 88 | 211.5 |
| Z044 | M | 44 | 0 | IgM ZIKV | 0.84932 | 0.11193 | 1.9223 | 0.11609 | 17 | 3.6 |
|  |  |  |  | IgG ZIKV | 0.76076 | 1.2406 | 0.88309 | 0.04352 | 20 | 7.3 |
|  |  |  |  | IgG ZEDIII | 0.30233 | 2.0128 | 0.18143 | 0.0023536 | 77 | 3.7 |
|  |  |  |  | SN ZIKV | 0.39419 | 0.001567 | 3.1241 | 0.023315 | 134 | 304.5 |
| Z045 | F | 32 | 0 | IgM ZIKV | 0.90483 | 0.033149 | 2.8807 | 0.19221 | 15 | 4.5 |
|  |  |  |  | IgG ZIKV | 0.89363 | 0.6159 | 1.1104 | 0.050623 | 22 | 6.3 |
|  |  |  |  | IgG ZEDIII | 0.9285 | 0.10269 | 0.83414 | 0.0089983 | 93 | 2.0 |
|  |  |  |  | SN ZIKV | 0.81188 | 1.4773 | 0.87795 | 0.0077781 | 113 | 38.9 |
| Z046 | M | 35 | 1 | IgM ZIKV | 0.95121 | 0.020356 | 3.1103 | 0.22869 | 14 | 3.0 |
|  |  |  |  | IgG ZIKV | 0.9616 | 3.779 | 0.46801 | 0.027418 | 17 | 8.9 |
|  |  |  |  | IgG ZEDIII | 0.9287 | 0.13936 | 0.8489 | 0.0072951 | 116 | 3.4 |
|  |  |  |  | SN ZIKV | 0.57779 | 4.9224 | 0.68383 | 0.0046819 | 146 | 75.1 |
| Z047 | F | 41 | 1 | IgM ZIKV | 0.91546 | 0.016107 | 3.1161 | 0.20064 | 16 | 3.7 |
|  |  |  |  | IgG ZIKV | 0.88359 | 0.6058 | 1.0593 | 0.035819 | 30 | 7.6 |
|  |  |  |  | IgG ZEDIII | 0.93405 | 0.034611 | 1.2336 | 0.0081494 | 151 | 4.9 |
|  |  |  |  | SN ZIKV | 0.60922 | 0.86261 | 1.2509 | 0.0075445 | 166 | 147.6 |
| Z048 | F | 35 | 0 | IgM ZIKV | 0.87399 | 0.37151 | 1.1539 | 0.056202 | 21 | 3.8 |
|  |  |  |  | IgG ZIKV | 0.83131 | 0.3075 | 1.0857 | 0.049027 | 22 | 3.0 |
|  |  |  |  | IgG ZEDIII | 0.39849 | 0.27832 | 0.39874 | 0.0031525 | 126 | 1.3 |
|  |  |  |  | SN ZIKV | 0.50424 | 2.3162 | 0.88078 | 0.0075716 | 116 | 63.3 |

Supplementary Table 2. Individual parameters from Wood’s equation and hidden data for all patients

ND: not determined, immune scar 0 = without, immune scar 1 = with
