# Supplementary material for: Zika virus-specific and orthoflavivirus-cross-reactive IgGs correlate with Zika virus seroneutralization depending on prior dengue virus infection
Source: PLoS Negl Trop Dis. 2025 Jul 9;19(7):e0013274. doi: 10.1371/journal.pntd.0013274 (PMC12240325; doi:10.1371/journal.pntd.0013274)
Supplement: S3 Table — (DOCX) [file pntd.0013274.s005.docx]

|  | All patients | | | | Patients with anamnestic response | | | | Patients without anamnestic response | | | |
| --- | --- | --- | --- | --- | --- | --- | --- | --- | --- | --- | --- | --- |
|  | **Day max  ZIKV-IgM** | **Day max ZIKV-IgG** | **Day max ZEDIII-IgG** | **Day max ZIKV-SN** | **Day max  ZIKV-IgM** | **Day max ZIKV-IgG** | **Day max ZEDIII-IgG** | **Day max ZIKV-SN** | **Day max  ZIKV-IgM** | **Day max ZIKV-IgG** | **Day max ZEDIII-IgG** | **Day max ZIKV-SN** |
| Number of values | 33 | 32 | 22 | 27 | 9 | 9 | 8 | 6 | 24 | 23 | 14 | 21 |
|  |  |  |  |  |  |  |  |  |  |  |  |  |
| Minimum | 12 | 12 | 39 | 8 | 12 | 13 | 39 | 8 | 12 | 12 | 64 | 19 |
| 5% Percentile | 12 | 13 | 41 | 12 | 12 | 13 | 39 | 8 | 12 | 13 | 64 | 19 |
| Median | 18 | 30 | 114 | 85 | 16 | 24 | 96 | 78 | 19 | 32 | 119 | 85 |
| 95% Percentile | 29 | 57 | 218 | 328 | 25 | 67 | 191 | 166 | 32 | 52 | 222 | 385 |
| Maximum | 34 | 67 | 222 | 404 | 25 | 67 | 191 | 166 | 34 | 52 | 222 | 404 |
| Range | 22 | 55 | 183 | 396 | 13 | 54 | 152 | 158 | 22 | 40 | 158 | 385 |

Supplementary Table 3. Distribution of Day_max_ of all patients, patients with an anamnestic response, and patients without an anamnestic response
