# Supplementary material for: Zika virus-specific and orthoflavivirus-cross-reactive IgGs correlate with Zika virus seroneutralization depending on prior dengue virus infection
Source: PLoS Negl Trop Dis. 2025 Jul 9;19(7):e0013274. doi: 10.1371/journal.pntd.0013274 (PMC12240325; doi:10.1371/journal.pntd.0013274)
Supplement: S4 Table — The Spearman r values are shown to the right of the diagonal and the p-value and number of individuals to the left. Bold underlined r values are those with a significant p-value highlighted in green. (DOCX) [file pntd.0013274.s006.docx]

A

| **p; n\ r** | Day max  ZIKV-IgM | | Level max  ZIKV-IgM | | Day max  ZIKV-IgG | | Level max ZIKV-IgG | | Day max  ZEDIII-IgG | | Level max  ZEDIII-IgG | | Day max  ZIKV-SN | | Level max  ZIKV-SN | |
| --- | --- | --- | --- | --- | --- | --- | --- | --- | --- | --- | --- | --- | --- | --- | --- | --- |
| Day max IgM ZIKV |  |  | 0,12 | | **0,43** | | -0,15 | | -0,11 | | -0,40 | | -0,08 | | -0,06 | |
| Level max IgM ZIKV | 0,51 | ; 33 |  |  | -0,06 | | **0,49** | | **0,50** | | -0,07 | | -0,08 | | 0,15 | |
| Day max IgG ZIKV | 0,01 | ; 32 | 0,73 | ; 32 |  |  | 0,13 | | -0,33 | | -0,07 | | 0,06 | | 0,09 | |
| Level max IgG ZIKV | 0,41 | ; 32 | <0.01 | ; 32 | 0,49 | ; 32 |  |  | 0,09 | | 0,05 | | -0,29 | | 0,26 | |
| Day max IgG ZEDIII | 0,62 | ; 22 | 0,02 | ; 22 | 0,14 | ; 22 | 0,69 | ; 22 |  |  | 0,19 | | **0,56** | | 0,44 | |
| Level max IgG ZEDIII | 0,07 | ; 22 | 0,74 | ; 22 | 0,77 | ; 22 | 0,82 | ; 22 | 0,40 | ; 22 |  |  | 0,21 | | **0,80** | |
| Day max SN ZIKV | 0,69 | ; 27 | 0,68 | ; 27 | 0,78 | ; 26 | 0,15 | ; 26 | 0,01 | ; 18 | 0,40 | ; 18 |  |  | 0,10 | |
| Level max SN ZIKV | 0,76 | ; 27 | 0,44 | ; 27 | 0,65 | ; 26 | 0,21 | ; 26 | 0,07 | ; 18 | <0.01 | ; 18 | 0,62 | ; 27 |  |  |

B

| **p; n\ r** | Day max  ZIKV-IgM | | Level max  ZIKV-IgM | | Day max  ZIKV-IgG | | Level max ZIKV-IgG | | Day max  ZEDIII-IgG | | Level max  ZEDIII-IgG | | Day max  ZIKV-SN | | Level max  ZIKV-SN | |
| --- | --- | --- | --- | --- | --- | --- | --- | --- | --- | --- | --- | --- | --- | --- | --- | --- |
| Day max IgM ZIKV |  |  | 0,01 | | **0,56** | | -0,05 | | -0,17 | | -0,34 | | -0,03 | | -0,08 | |
| Level max IgM ZIKV | 0,95 | ; 24 |  |  | -0,08 | | **0,55** | | 0,40 | | 0,19 | | 0,02 | | 0,26 | |
| Day max IgG ZIKV | 0,01 | ; 23 | 0,73 | ; 23 |  |  | 0,20 | | -0,39 | | -0,28 | | 0,09 | | -0,17 | |
| Level max IgG ZIKV | 0,81 | ; 23 | <0.01 | ; 23 | 0,37 | ; 23 |  |  | 0,14 | | 0,02 | | -0,19 | | 0,35 | |
| Day max IgG ZEDIII | 0,55 | ; 14 | 0,16 | ; 14 | 0,17 | ; 14 | 0,62 | ; 14 |  |  | 0,49 | | **0,60** | | **0,76** | |
| Level max IgG ZEDIII | 0,24 | ; 14 | 0,52 | ; 14 | 0,32 | ; 14 | 0,95 | ; 14 | 0,08 | ; 14 |  |  | 0,34 | | **0,79** | |
| Day max SN ZIKV | 0,91 | ; 21 | 0,93 | ; 21 | 0,71 | ; 20 | 0,43 | ; 20 | 0,04 | ; 13 | 0,26 | ; 13 |  |  | 0,15 | |
| Level max SN ZIKV | 0,72 | ; 21 | 0,25 | ; 21 | 0,48 | ; 20 | 0,13 | ; 20 | <0.01 | ; 13 | <0.01 | ; 13 | 0,53 | ; 21 |  |  |

C

| **p; n\ r** | Day max  ZIKV-IgM | | Level max  ZIKV-IgM | | Day max  ZIKV-IgG | | Level max ZIKV-IgG | | Day max  ZEDIII-IgG | | Level max  ZEDIII-IgG | | Day max  ZIKV-SN | | Level max  ZIKV-SN | |
| --- | --- | --- | --- | --- | --- | --- | --- | --- | --- | --- | --- | --- | --- | --- | --- | --- |
| Day max ZIKV-IgM |  |  | 0,15 | | -0,09 | | -0,62 | | -0,07 | | -0,49 | | 0,20 | | -0,03 | |
| Level max ZIKV-IgM | 0,70 | ; 9 |  |  | -0,03 | | 0,02 | | 0,57 | | -0,48 | | -0,14 | | -0,37 | |
| Day max ZIKV-IgG | 0,82 | ; 9 | 0,95 | ; 9 |  |  | -0,05 | | -0,33 | | 0,52 | | 0,26 | | **0,89** | |
| Level max ZIKV-IgG | 0,08 | ; 9 | 0,98 | ; 9 | 0,91 | ; 9 |  |  | -0,60 | | 0,14 | | -0,71 | | -0,31 | |
| Day max ZEDIII-IgG | 0,87 | ; 8 | 0,15 | ; 8 | 0,43 | ; 8 | 0,13 | ; 8 |  |  | -0,19 | | 0,50 | | 0,00 | |
| Level max ZEDIII-IgG | 0,22 | ; 8 | 0,24 | ; 8 | 0,20 | ; 8 | 0,75 | ; 8 | 0,66 | ; 8 |  |  | -0,30 | | 0,90 | |
| Day max ZIKV-SN | 0,71 | ; 6 | 0,80 | ; 6 | 0,66 | ; 6 | 0,14 | ; 6 | 0,45 | ; 5 | 0,68 | ; 5 |  |  | -0,09 | |
| Level max ZIKV-SN | 1,00 | ; 6 | 0,50 | ; 6 | 0,03 | ; 6 | 0,56 | ; 6 | 1,00 | ; 5 | 0,08 | ; 5 | 0,92 | ; 6 |  |  |

Supplementary table 4. Spearman correlation matrix for patients: A) the whole cohort, B) patients without a flavivirus anamnestic response, and C) patients with a flavivirus anamnestic response. The Spearman r values are shown to the right of the diagonal and the p-value and number of individuals to the left. Bold underlined r values are those with a significant p-value highlighted in green.
