# Supplementary material for: Zika virus-specific and orthoflavivirus-cross-reactive IgGs correlate with Zika virus seroneutralization depending on prior dengue virus infection
Source: PLoS Negl Trop Dis. 2025 Jul 9;19(7):e0013274. doi: 10.1371/journal.pntd.0013274 (PMC12240325; doi:10.1371/journal.pntd.0013274)
Supplement: S5 Table — The positive threshold ODr for IgM and IgG against ZIKV were 3. The positive threshold ODrs for IgG to ZEDIII, D1EDIII, D2EDIII, D3EDIII, and D4EDIII were 1.54, 1.92, 1.52, 1.86, and 1.89, respectively. The positive threshold titer of SN was 1/40. (DOCX) [file pntd.0013274.s007.docx]

A

|  | **ZEDIII-IgG** | **D1EDIII-IgG** | **D2EDIII-IgG** | **D3EDIII-IgG** | **D4EDIII-IgG** | **ZIKV-SN** | **DENV1-SN** | **DENV2-SN** | **DENV3-SN** | **DENV4-SN** |
| --- | --- | --- | --- | --- | --- | --- | --- | --- | --- | --- |
| Number of values | 23 | 22 | 24 | 22 | 24 | 24 | 23 | 23 | 23 | 23 |
|  |  |  |  |  |  |  |  |  |  |  |
| Minimum | 1.3 | 0.8 | 0.9 | 0.8 | 0.9 | 20 | 40 | 40 | 20 | 20 |
| 5% Percentile | 1.4 | 0.8 | 0.9 | 0.8 | 1 | 20 | 40 | 48 | 24 | 20 |
| Median | 3 | 1.2 | 1.4 | 1.1 | 1.4 | 80 | 160 | 160 | 80 | 40 |
| 95% Percentile | 8.7 | 4.6 | 4.5 | 5 | 2.7 | 320 | 320 | 320 | 320 | 160 |
| Maximum | 8.8 | 5 | 4.7 | 5.4 | 2.8 | 320 | 320 | 320 | 320 | 160 |
| Range | 7.4 | 4.2 | 3.8 | 4.6 | 1.9 | 300 | 280 | 280 | 300 | 140 |
| Positives (%) | 96 | 9 | 12 | 14 | 25 | 92 | 100 | 100 | 96 | 78 |

B

|  | **ZEDIII-IgG** | **D1EDIII-IgG** | **D2EDIII-IgG** | **D3EDIII-IgG** | **D4EDIII-IgG** | **ZIKV-SN** | **DENV1-SN** | **DENV2-SN** | **DENV3-SN** | **DENV4-SN** |
| --- | --- | --- | --- | --- | --- | --- | --- | --- | --- | --- |
| Number of values | 6 | 8 | 9 | 9 | 9 | 8 | 8 | 8 | 8 | 8 |
|  |  |  |  |  |  |  |  |  |  |  |
| Minimum | 2.1 | 1 | 1 | 0.96 | 1.1 | 20 | 40 | 160 | 160 | 20 |
| 5% Percentile | 2.1 | 1 | 1 | 0.96 | 1.1 | 20 | 40 | 160 | 160 | 20 |
| Median | 3.2 | 1.3 | 1.6 | 1.2 | 1.8 | 80 | 240 | 320 | 240 | 60 |
| 95% Percentile | 5.5 | 4.1 | 2.8 | 4.1 | 4.7 | 320 | 320 | 320 | 320 | 320 |
| Maximum | 5.5 | 4.1 | 2.8 | 4.1 | 4.7 | 320 | 320 | 320 | 320 | 320 |
| Range | 3.5 | 3 | 1.7 | 3.2 | 3.6 | 300 | 280 | 160 | 160 | 300 |
| Positives (%) | 100 | 13 | 22 | 11 | 44 | 88 | 100 | 100 | 100 | 63 |

Supplementary Table 5. A) Patients at day 28 without a previous DENV infection B) Patients at day 28 with a previous DENV infection. The positive threshold ODr for IgM and IgG against ZIKV were 3. The positive threshold ODrs for IgG to ZEDIII, D1EDIII, D2EDIII, D3EDIII, and D4EDIII were 1.54, 1.92, 1.52, 1.86, and 1.89, respectively. The positive threshold titer of SN was 1/4
